# Knowing friend from foe

**DOI:** 10.7554/eLife.83121

**Published:** 2022-10-07

**Authors:** Magnus Hallas-Møller, Katja S Johansen

**Affiliations:** 1 Department of Geosciences and Natural Resource Management, Copenhagen University Copenhagen Denmark

**Keywords:** allorecognition, polysaccharide monooxygenase, somatic cell fusion, non self recognition, cell fusion checkpoint, chitin, Neurospora

## Abstract

How does a protein at the cell wall determine if a newly encountered fungus is safe to fuse with?

**Related research article** Detomasi TC, Ramírez AMR, Sayler RI, Gonçalves AP, Marletta MA, Glass NL. 2022. A moonlighting function of a chitin polysaccharide monooxygenase, CWR-1, in *Neurospora crassa* allorecognition. *eLife* **11**:e80459. doi: 10.7554/eLife.80459.

Many multicellular organisms have mechanisms in place that allow them to detect which cells belong to them, and which cells are from another organism. Being able to discriminate self from non-self, known as allorecognition, is vital for the sustainability of life. For instance, our immune system uses this mechanism to identify and attack non-self cells and tissues, which is why our bodies sometimes reject transplanted organs donated from someone else (Callemeyn et al., 2022).

Filamentous fungi – which are made up of microscopic thread-like structures called hypha – also rely on allorecognition to decide what to do when they come across hyphae from other fungi. Merging with other hyphae would allow the fungus to expand its network and access more resources that may benefit its survival. However, before this can happen, the fungus uses allorecognition to determine if a newly encountered hypha is safe to fuse to.

In the fungus *Neurospora crassa,* this process of allorecognition involves three checkpoints (Gonçalves et al., 2020; Zhao et al., 2015). First, the hyphae release chemical signals that attract fusion compatible hyphae (Heller et al., 2016). Second, two proteins called CWR-1 and CWR-2 determine whether the cell wall surrounding the hyphae will dissolve so the cells can merge their membranes and mix their cytoplasmic content (Gonçalves et al., 2019). Once the fungi fuse, final checks are carried out, with the failure of these tests triggering the death of the newly joined hypha. Now, in eLife, Louise Glass and co-workers from the University of California, Berkeley – including Tyler Detomasi and Adriana Rico-Ramírez as joint first authors – report new insights into how the CWR-1 protein regulates the second checkpoint of allorecognition (Detomasi et al., 2022).

*N. crassa* have different versions, or alleles, of the genes that encode CWR-1 and CWR-2, and these can be divided in to six different ‘haplogroups’ based on their degree of similarity (Gonçalves et al., 2019). Only *N. crassa* with CWR-1 and CWR-2 proteins from the same haplogroup can fuse: if the gene for CWR-1 in one hypha is in a different haplogroup to the gene for CWR-2 in the other, their cells walls will remain intact and their membranes will not merge (Figure 1). This shows that variations in these two proteins determine whether or not hyphae are compatible for fusion.

**Figure 1.**
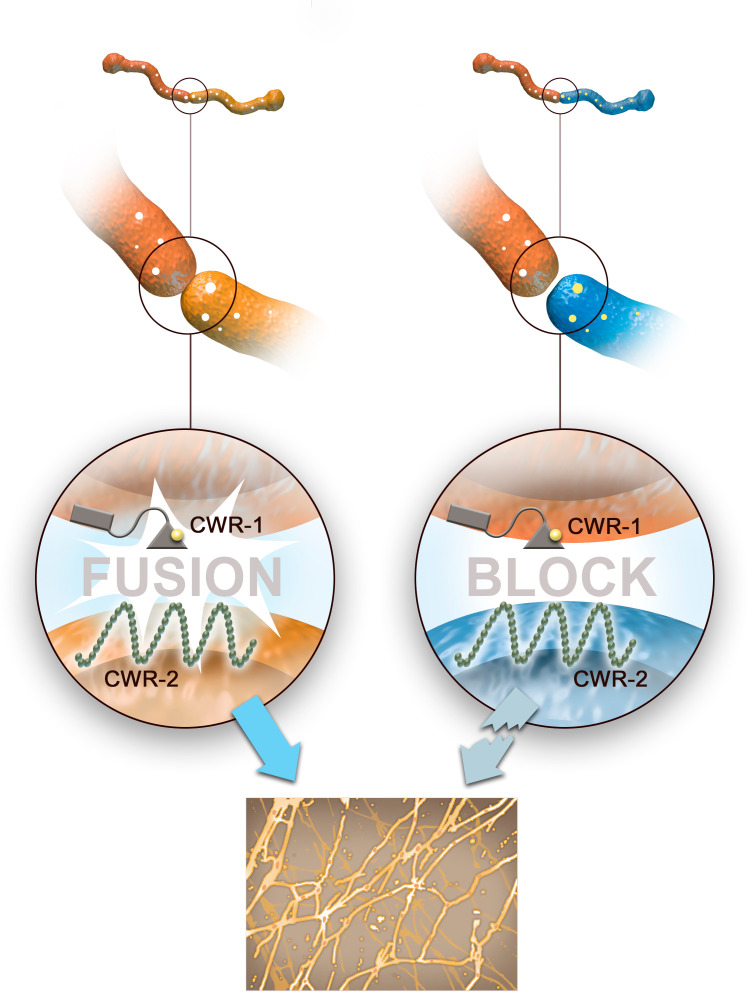
The second checkpoint of allorecognition in filamentous fungi. When hyphae from two distinct fungi come into contact, a series of checkpoints are initiated to make sure the fungi are genetically compatible. The second stage of this allorecognition process is regulated by two proteins at the cell wall called CWR-1 and CWR-2. If the two hyphae contain CWR-1 and CWR-2 proteins from the same haplogroup (left), the hyphae dissolve their cells walls, merge their membranes, and mix their cytoplasmic content together. This allows the fungi to expand their network of interconnected hyphae (bottom panel). If the two hyphae contain CWR-1 and CWR-2 proteins from different haplogroups (right), the cell wall does not dissolve and the genetically incompatible hyphae cannot proceed with fusion.

Image credit: Henning Dalhoff.

Using well-established methods, Detomasi et al. revealed that the CWR-1 protein is part of a family of copper-containing enzymes called lytic polysaccharide monooxygenases, or LPMOs for short. Similar to LPMOs found in other fungal species, the CWR-1 proteins from all six haplogroups degrade the polysaccharide chitin, a long-chain carbohydrate that maintains the structure of the cell wall and helps anchor other cell wall components in place (Brown et al., 2019). This enzymatic activity depends on the copper in the protein, which is coordinated by two amino acids in what is known known as a histidine brace (Ipsen et al., 2021). Surprisingly, Detomasi et al. found that mutating the histidine brace of CWR-1 did not stop *N. crassa* strains from exhibiting normal allorecognition and only fusing with genetically compatible fungi, despite the enzyme being inactive.

Through a series of clever genetic mutations, Detomasi et al. found that CWR-1 does not need its enzymatic activity or the domains of the protein that bind to chitin to carry out its role in allorecognition. It does, however, require its catalytic domain. Further mutations showed that modifying regions in the catalytic domain of CWR-1 that are predicted to interact with chitin (but are not responsible for the protein’s enzymatic activity) altered which *N. crassa* strains could fuse their hyphae together. This suggests that these sections of the CWR-1 protein confer the allele specificity needed for cells to pass the second checkpoint of allorecognition.

To our knowledge, this is the first time a LPMO protein has been shown to have a function that does not involve the degradation of polysaccharides. However, LPMO-like proteins which do not catalyze the breakdown of carbohydrates have been found in other fungal species (Garcia-Santamarina et al., 2020; Labourel et al., 2020). While these proteins look like LMPOs based on their amino acid sequence, a closer inspection reveals that their copper-binding sites are slightly different than expected. These LPMO-like proteins have been shown to be important for copper import in the fungal species *Cryptococcus neoformans,* and for establishing a symbiosis relationship between the fungus *Laccaria bicolor* and plant roots.

This work is a major step towards understanding allorecognition in fungi, but several questions remain. As Detomasi et al. point out, future work is needed to probe how CWR-1 and CWR-2 mechanically block cell fusion. Furthermore, it is still unclear if and how CWR-1, which binds to chitin in the cell wall, gets in to contact with the CWR-2 protein on the membrane of the neighboring hypha despite there being two layers of cell wall between them. Finally, while initial investigations suggest that the CWR-1/CWR-2 model likely occurs in other species (Gonçalves et al., 2019), it is still uncertain how widespread this mechanism is across the fungal kingdom.

## References

[bib1] Brown HE, Esher SK, Alspaugh JA, Latgé JP (2019). The Fungal Cell Wall.

[bib2] Callemeyn J, Lamarthée B, Koenig A, Koshy P, Thaunat O, Naesens M (2022). Allorecognition and the spectrum of kidney transplant rejection. Kidney International.

[bib3] Detomasi TC, Ramírez AMR, Sayler RI, Gonçalves AP, Marletta MA, Glass NL (2022). A moonlighting function of a chitin polysaccharide monooxygenase, CWR-1, in *Neurospora crassa* allorecognition. eLife.

[bib4] Garcia-Santamarina S, Probst C, Festa RA, Ding C, Smith AD, Conklin SE, Brander S, Kinch LN, Grishin NV, Franz KJ, Riggs-Gelasco P, Lo Leggio L, Johansen KS, Thiele DJ (2020). A lytic polysaccharide monooxygenase-like protein functions in fungal copper import and meningitis. Nature Chemical Biology.

[bib5] Gonçalves AP, Heller J, Span EA, Rosenfield G, Do HP, Palma-Guerrero J, Requena N, Marletta MA, Glass NL (2019). Allorecognition upon fungal cell-cell contact determines social cooperation and impacts the acquisition of multicellularity. Current Biology.

[bib6] Gonçalves AP, Heller J, Rico-Ramírez AM, Daskalov A, Rosenfield G, Glass NL (2020). Conflict, competition, and cooperation regulate social interactions in filamentous fungi. Annual Review of Microbiology.

[bib7] Heller J, Zhao J, Rosenfield G, Kowbel DJ, Gladieux P, Glass NL (2016). Characterization of greenbeard genes involved in long-distance kind discrimination in a microbial eukaryote. PLOS Biology.

[bib8] Ipsen JØ, Hallas-Møller M, Brander S, Lo Leggio L, Johansen KS (2021). Lytic polysaccharide monooxygenases and other histidine-brace copper proteins: structure oxygen activation and biotechnological applications. Biochemical Society Transactions.

[bib9] Labourel A, Frandsen KEH, Zhang F, Brouilly N, Grisel S, Haon M, Ciano L, Ropartz D, Fanuel M, Martin F, Navarro D, Rosso M-N, Tandrup T, Bissaro B, Johansen KS, Zerva A, Walton PH, Henrissat B, Leggio LL, Berrin J-G (2020). A fungal family of lytic polysaccharide monooxygenase-like copper proteins. Nature Chemical Biology.

[bib10] Zhao J, Gladieux P, Hutchison E, Bueche J, Hall C, Perraudeau F, Glass NL (2015). Identification of allorecognition loci in *Neurospora crassa* by genomics and evolutionary approaches. Molecular Biology and Evolution.

